# Supplementary material for: Plasmatic Inactive IL-18 Predicts a Worse Overall Survival for Advanced Non-Small-Cell Lung Cancer with Early Metabolic Progression after Immunotherapy Initiation
Source: Cancers (Basel). 2024 Jun 14;16(12):2226. doi: 10.3390/cancers16122226 (PMC11202099; doi:10.3390/cancers16122226)
Supplement: Supplementary file 1 [file cancers-16-02226-s001.zip › cancers-2985589-supplementary.pdf]

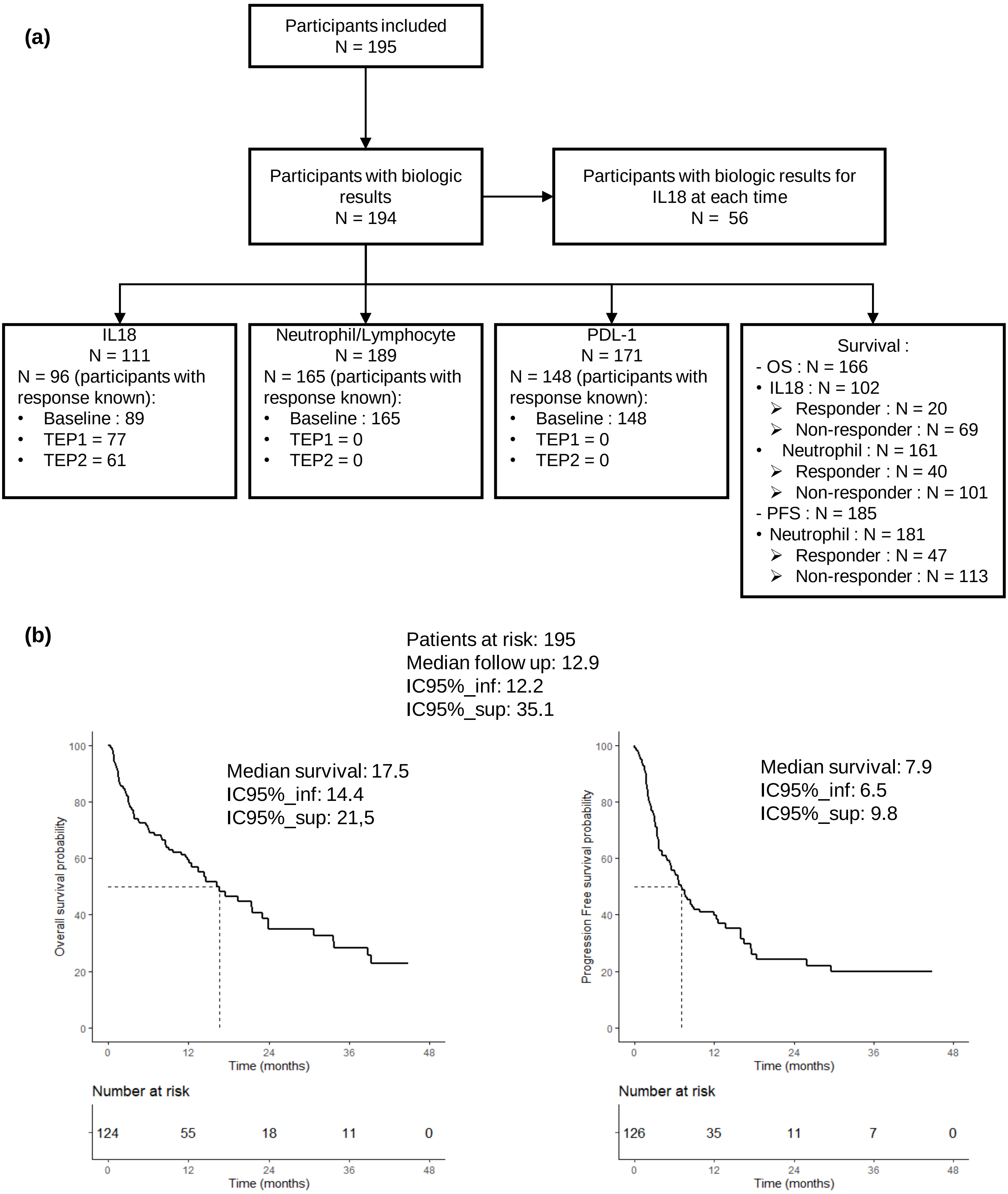

Supplementary Figure S1: Cohort characterization. (a) Schematic diagram illutrating the patient flow chart. **(b)** Overall Survival (OS) probability (left panel) and Progression Free Survival (PFS) probability (righ panel). IC: Interval confidence

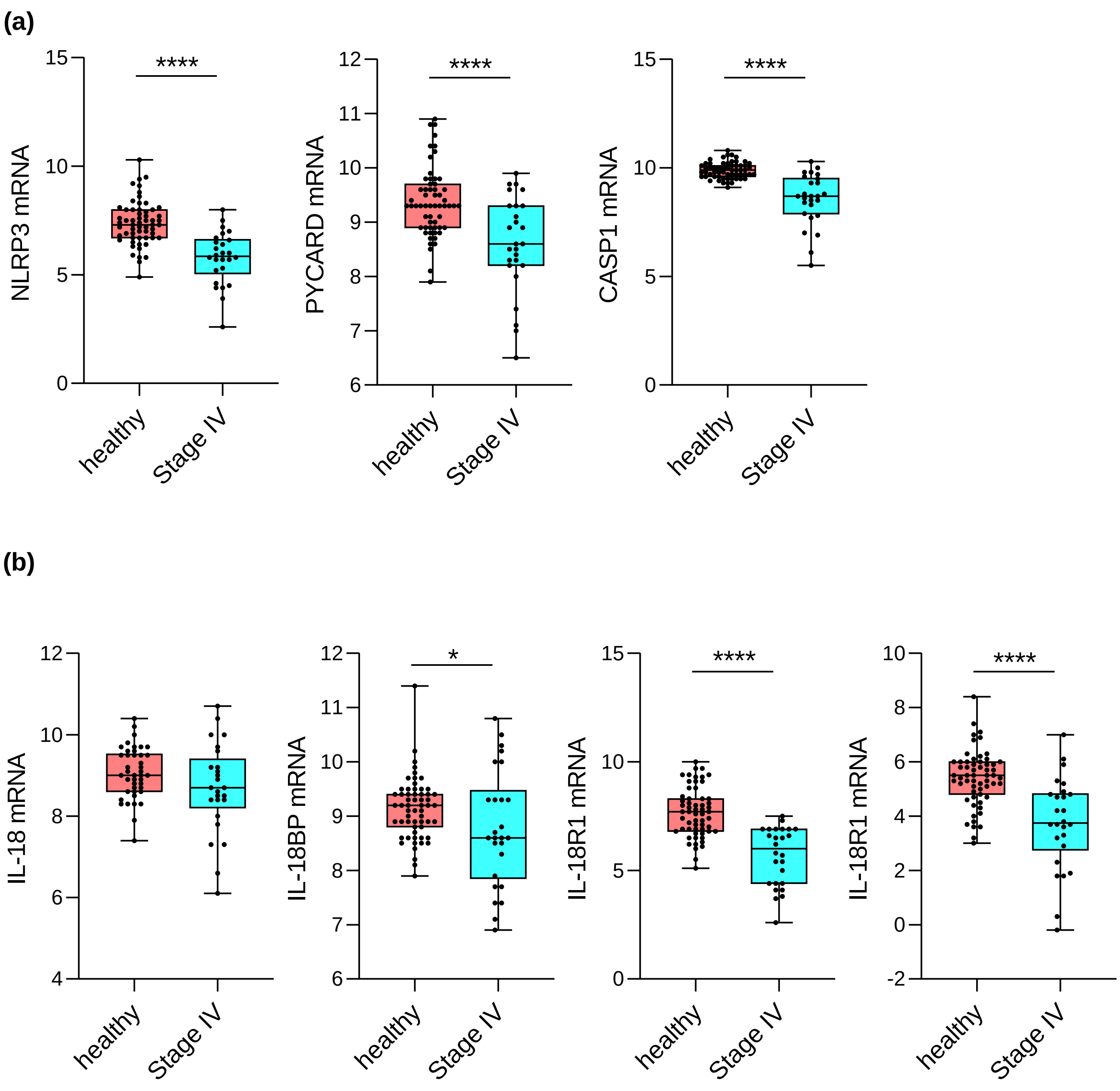

Supplementary Figure S2: mRNA expression in NSCLC patients.
(a) mRNA expression of NLRP3, PYCARD and CASP1 or **(b)** IL18, IL18BP, IL18R1 and IL18RAP between healthy controls (healthy) and NSCLC stage IV patients from 565 individuals, corresponding to 59 controls and 26 NSCLC stage IV patients. P-values were determined by Two-tailed unpaired t-test with Welch correction. $*p < 0.05$, $****p < 0.0001$.

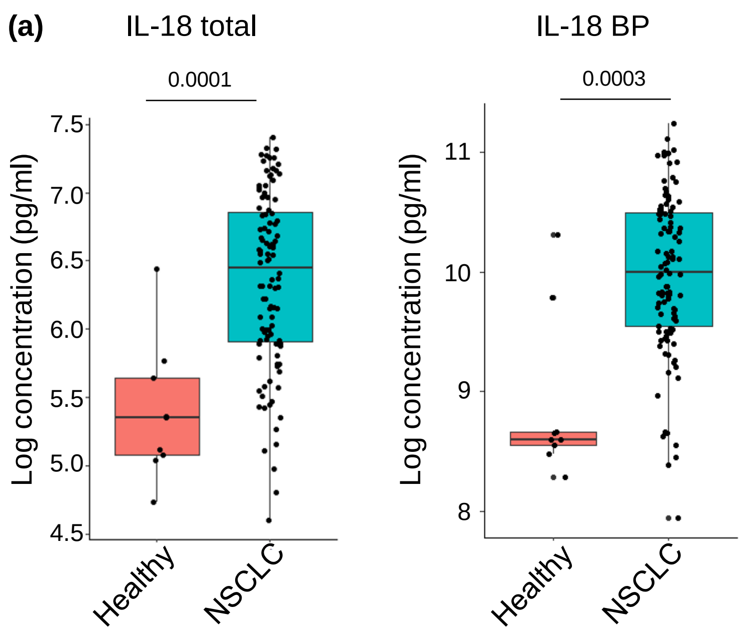

Supplementary Figure S3. (a). The concentration of total IL-18 and IL-18BP were measured in plasma samples. Bars show median levels. Each point represents one sample. p-values were determined by wilcoxom rank sum test with continuity correction.

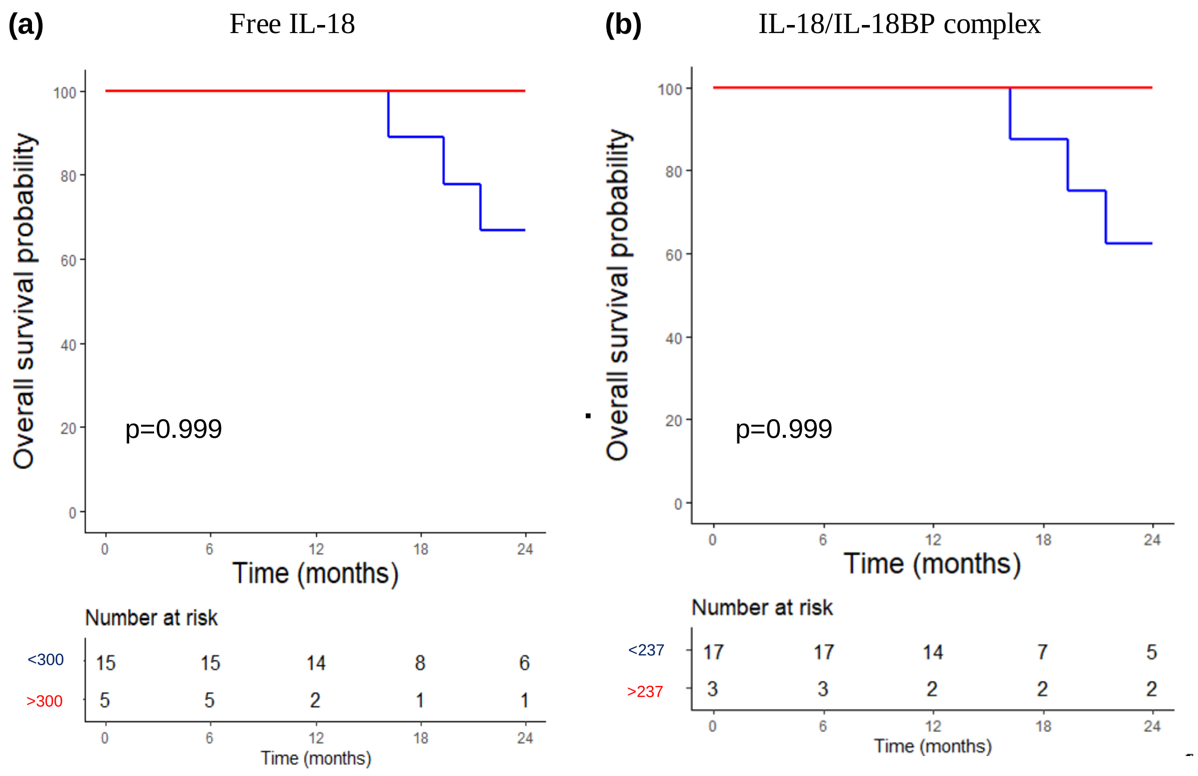

Supplementary Figure S4: Overall survival in ICI responding patients

Survival analyses (Kaplan–Meier). (**a**) Free IL-18, (**b**) IL-18/IL-18 complex. Blue lines correspond to concentrations inferior at cut off. Red lines correspond to concentrations superior at cut off. Number at risk with censored data are shown. P values were determined by Mentel Cox test.

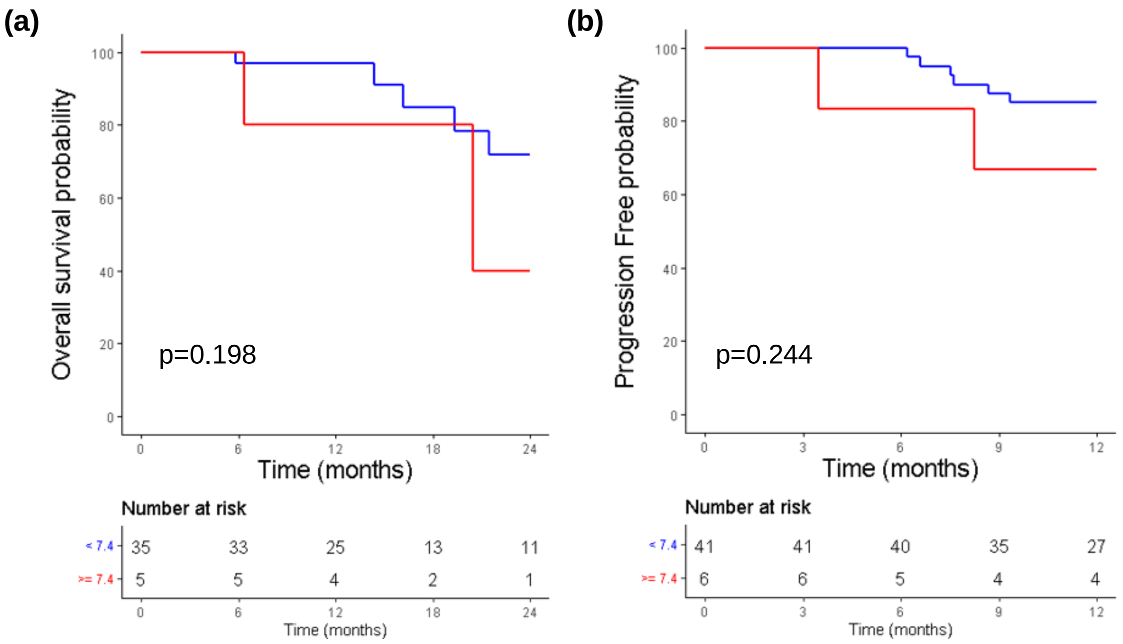

Supplementary Figure S5: Impact of neutrophil concentration in ICI responding patients. Survival analyses (Kaplan–Meier). **(a)** Overall survival, **(b)** Progression free survival. Blue lines correspond to concentrations inferior at cut off. Red lines correspond to concentrations superior at cut off. Number at risk with censored data are shown. p values were determined by Mentel Cox test.

Supplementary Table S1: IL-18 related compound concentrations within responding and non-responding groups (normalized data)

Analyte	Non-responding Moy (SD), Nobs	Responding Moy (SD), Nobs	p-value	FDR
Baseline				
IL-18 total	6.37 (0.64) /69	6.39 (0.6) /20	0.93	1
IL-18 BP	9.98 (0.6) /69	9.95 (0.68) /20	0.97	1
IL-18/IL-18BP Complex	4.65 (2.36) /69	4.39 (1.95) /20	0.69	1
IL-18 free	5.07 (0.92) /69	5.05 (1.01) /20	0.93	1
TEP1				
IL-18 total	6.5 (0.74) /57	6.26 (0.64) /20	0.204	0.612
IL-18 BP	10.06 (0.57) /57	10.13 (0.59) /20	0.634	1
IL-18/IL-18BP Complex	4.46 (2.97) /57	2.96 (3.6) /20	0.071 TREND	0.18
IL-18 free	5.04 (1.05) /57	4.81 (1.29) /20	0.42	1
TEP2				
IL-18 total	6.37 (0.72) /42	6.21 (0.65) /19	0.418	1
IL-18 BP	10.07 (0.58) /42	10.08 (0.79) /19	0.942	1
IL-18/IL-18BP Complex	4.18 (3.09) /42	3.51 (3.28) /19	0.447	1
IL-18 free	4.93 (1.22) /42	4.44 [(1.83) /19	0.22	0.66

Supplementary Table S2: Evolution of IL-18 related compound concentrations during ICI treatment

Steps of treatment	Test	p-value	FDR
**IL-18 total**	All patients		
Baseline vs TEP 1	Paired Wilcoxon	0.77	1
Baseline vs TEP 2	Paired Wilcoxon	0.13	0.39
TEP 1 vs TEP 2	Paired Wilcoxon	0.01	0.035
	Responding		
Baseline vs TEP 1	Paired T test	0.54	1
Baseline vs TEP 2	Paired T test	0.099	0.29
TEP 1 vs TEP 2	Paired T test	0.14	0.43
	Non-responding		
Baseline vs TEP 1	Paired Wilcoxon	0.78	1
Baseline vs TEP 2	Paired Wilcoxon	0.1	0.31
TEP 1 vs TEP 2	Paired T test	0.17	0.51
**IL-18BP**	All patients		
Baseline vs TEP 1	Paired T test	0.20098	0.602940
TEP 1 vs TEP 2	Paired T test	0.772231	1.000000
TEP 2 vs TEP 3	Paired T test	0.131094	0.393282
	Responding		
Baseline vs TEP 1	Paired T test	0.615625	1
TEP 1 vs TEP 2	Paired T test	0.710627	1
TEP 2 vs TEP 3	Paired T test	0.337827	1
	Non-responding		
Baseline vs TEP 1	Paired Wilcoxon	0.52817	1
Baseline vs TEP 2	Paired T test	0.337827	1
TEP 1 vs TEP 2	Paired Wilcoxon	0.89993	1
**IL-18/IL-18BP complex**	All patients		
Baseline vs TEP 1	Paired Wilcoxon	0.37516	1
Baseline vs TEP 2	Paired Wilcoxon	0.743525	1
TEP 1 vs TEP 2	Paired Wilcoxon	0.38695	1
	Responding		
Baseline vs TEP 1	Paired Wilcoxon	0.46375	1
Baseline vs TEP 2	Paired Wilcoxon	0.375458	1
TEP 1 vs TEP 2	Paired Wilcoxon	0.75361	1
	Non-responding		
Baseline vs TEP 1	Paired Wilcoxon	0.06074	0.182220
Baseline vs TEP 2	Paired Wilcoxon	0.248201	0.744603
TEP 1 vs TEP 2	Paired Wilcoxon	0.59612	1.000000
**IL-18 free**	All patients		
Baseline vs TEP 1	Paired Wilcoxon	0.36308	1.0000
Baseline vs TEP 2	Paired Wilcoxon	0.0488	0.146538
TEP 1 vs TEP 2	Paired Wilcoxon	0.058	0.176940
	Responding		
Baseline vs TEP 1	Paired T test	0.61757	1.0000
Baseline vs TEP 2	Paired Wilcoxon	0.211426	0.634278
TEP 1 vs TEP 2	Paired Wilcoxon	0.2312	0.693600
	Non-responding		
Baseline vs TEP 1	Paired Wilcoxon)	0.42841	1.0000
Baseline vs TEP 2	Paired Wilcoxon)	0.136554	0.409662
TEP 1 vs TEP 2	Paired Wilcoxon)	0.6331	1.0000
